# Determination of Half-lives of Circulating FSH and LH Glycoforms in Women During GnRH Receptor Blockade

**DOI:** 10.1210/clinem/dgac434

**Published:** 2022-08-01

**Authors:** Leif Wide, Karin Eriksson, Patrick M Sluss, Janet E Hall

**Affiliations:** Department of Medical Sciences, Clinical Chemistry, University Hospital, SE 751 85 Uppsala, Sweden; Department of Medical Sciences, Clinical Chemistry, University Hospital, SE 751 85 Uppsala, Sweden; Pathology Service, Massachusetts General Hospital, Boston, MA 02114, USA; Reproductive Endocrine Unit, Department of Medicine, Massachusetts General Hospital, Boston, MA 02114, USA; Reproductive Endocrine Unit, Department of Medicine, Massachusetts General Hospital, Boston, MA 02114, USA; National Institute of Environmental Health Sciences, National Institutes of Health, Research Triangle Parc, NC 27709, USA

**Keywords:** GnRH antagonist, GnRH receptor blockade, half-life in circulation, FSH glycoforms, LH glycoforms

## Abstract

**Context:**

Both FSH and LH circulate as 2 glycoforms, differing in number of glycans: low-N-glycosylated glycoforms, FSHtri and LHdi, and fully N-glycosylated glycoforms, FSHtetra and LHtri.

**Objectives:**

To determine the half-lives of endogenous circulating gonadotropin glycoforms in women during GnRH receptor blockade.

**Design/Participants:**

Serum samples were collected in 8 healthy women before and up to 20 hours after administration of the NAL-GLU GnRH antagonist. Three women were in early follicular phase, 2 at mid-cycle phase, and 3 were postmenopausal.

**Main Outcome Measures:**

The half-life of each glycoform was estimated by monoexponential decay for FSH (n = 8) and LH (n = 5). Data were analyzed using paired *t* tests.

**Results:**

Half-lives in the circulation of low-N-glycosylated glycoforms of both FSH and LH were shorter than those of the fully N-glycosylated glycoforms (mean; range, FSHtri 343; 116-686 minutes vs FSHtetra 757; 436-1038, minutes, *P* = 0.0003; LHdi 125, 84-198 minutes vs LHtri 164, 107-235 minutes, *P* = 0.004). The half-lives of low-and fully N-glycosylated forms of LH were shorter than the corresponding half-lives of FSH glycoforms, *P* = 0.0008.

**Conclusions:**

For both FSH and LH, low-N-glycosylated glycoforms disappeared from the circulation faster than the fully N-glycosylated. The half-lives of low and fully N-glycosylated forms of LH were shorter than the corresponding half-lives of FSH. The estimated values for half-life in the circulation of total FSH and total LH will depend on the relative amounts of the 2 glycoforms of each hormone and their individual disappearance rates in circulation.

The human pituitary gonadotropins, FSH and LH, circulate in blood, displaying heterogeneous spectra of molecules. Both the FSH and LH molecules are heterodimers consisting of an alpha-subunit polypeptide, which is common to FSH, LH, and TSH, and of a noncovalently linked beta-subunit polypeptide that confers specificity. When alpha-subunits are glycosylated at each of their 2 N-glycosylation sites, they can combine with beta subunits having different degrees of N-glycosylation. There are 2 potential N-glycosylation sites on the FSH beta subunit and 1 on the LH beta subunit. FSH and LH circulate each as 2 glycoforms that are termed low-N-glycosylated, FSHtri and LHdi, and fully N-glycosylated, FSHtetra and LHtri, according to their total number of N-glycans per molecule (1). The glycoforms are further modulated by sulfonation and sialylation, together referred to as anionic monosaccharide (AMS) residues.

The disappearance rates of endogenous human FSH and LH from the circulation have been estimated during GnRH receptor blockade in women (2-6). However, the impact of low and fully N-glycosylated glycoforms on gonadotropin clearance was not investigated in these studies.

In the current study, we determined the disappearance rates of endogenous circulating low and fully N-glycosylated glycoforms of FSH (FSHtri and FSHtetra) and LH (LHdi and LHtri) during GnRH receptor blockade in women.

## Subjects and Methods

### Subjects and Experimental Design of the GnRH Receptor Blockade Study

Eight healthy women participated: 3 women were in the early follicular phase (EF), 2 at the gonadotropin midcycle surge (MC), and 3 were postmenopausal women (PM; defined as 2 years or more after the final menstrual period and off any estrogen replacement for at least 6 months). The principle underlying the use the GnRH receptor blockade model to assess the pharmacodynamics of endogenous FSH and LH has been described previously (2-6). The subjects were admitted to the General Clinical Research Center of the Massachusetts General Hospital. For each study, blood was sampled every 10 minutes for 4 hours before and 8 hours after subcutaneous administration of 150 µg/kg of the NAL-GLU GnRH antagonist, followed by hourly samples for an additional 12 hours. Samples were pooled for the following time periods: 4 to 2 hours before (basal level); and 2 to 4 hours (3-hour level), 6 to 8 hours (7-hour level), and 16 to 20 hours (18-hour level) after antagonist administration. The study was approved by the institutional review board of the Massachusetts General Hospital, and all subjects provided written informed consent.

## Analytical Methods

### Immunoassay of Serum FSH and LH

The concentrations of FSH and LH in serum samples and in separated fractions after electrophoreses were measured using time-resolved sandwich fluoroimmunoassays (Delfia, PerkinElmer-Wallac Oy, Turku, Finland) (7, 8). The methods permitted measurements of the hormones directly in the 0.075 M veronal (Sigma-Aldrich Chemie Gmbh, Germany) buffer at pH 8.7 eluted from electrophoreses. All sera were initially tested to identify and exclude individuals with the common variant form of LH as this variant is known to affect the disappearance of LH from the circulation (6, 9). Three women, 1 EF, 1 MC and 1 PM, were found to be heterozygous for the common variant form of LH and were therefore excluded from further LH analyses. Gonadotropin values were expressed in IU/L using the International Standards for Pituitary FSH (94/632) and LH (80/552) as reference standards. The detection limits were less than 0.02 IU/L serum and the interassay coefficient of variation was less than 3% for both hormones. The detection limit of the 2 hormones in fractions from electrophoresis was approximately 100 attograms.

### Frequency of Glycoforms of FSH and LH, and AMS Residues per Glycoform Molecule

All serum samples were analyzed with an electrophoresis technique using a 0.10% agarose suspension in veronal buffer at pH 8.7, as previously described (1). The motilities were expressed in relation to that of endogenous human serum albumin. The area of eluted gonadotropin was resolved into peaks at the positions for different numbers of AMS residues per molecule. The frequencies of the 2 glycoforms of FSH and of LH in serum samples and the median numbers of AMS residues per glycoform molecule were calculated from the electrophoretic distributions using the FSH and LH algorithms as described (1).

### Data Analyses

The half-lives of the gonadotropin glycoforms in the circulation during GnRH receptor blockade were determined after converting the values to percentages of the basal level. Because an increase in the LH concentration was observed in 3 of the 5 individuals between the 7-hour and the 18-hour samples, suggesting some degree of recovery from the effects of GnRH receptor blockade, only values up to 7 hours were used for calculation of the half-life of LH. All values were included for estimation of half-life of FSH. The half-lives were determined by 1-phase exponential decay as in previous studies (2-6). The half-life was calculated assuming a maximum percent inhibition from baseline of 95 for LHdi, 86 for LHtri, 70 for FSHtri, and 54 for FSHtetra, which were nadir values estimated from analyzing the decays of the different glycoforms. All values passed tests for normal distribution (the Shapiro-Wilk test and the Kolmogorov-Smirnov test). Paired *t* tests were used for comparisons of half-lives for low vs fully N-glycosylated glycoforms of FSH and LH, respectively.

## Results

### Serum Concentrations of Gonadotropin Glycoforms Before and During Blockade With GnRH Antagonist

**Figure 1.**
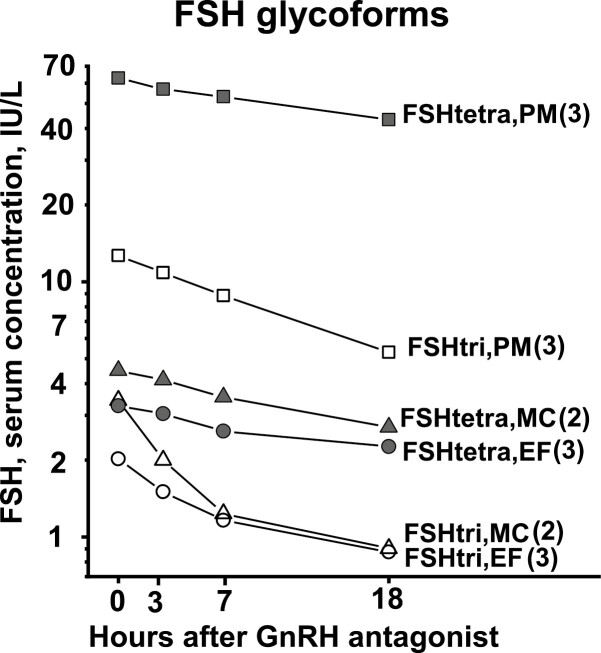
Mean changes of serum concentration of FSH glycoforms after GnRH-receptor antagonist, expressed in IU/L, for women in early follicular phase (EF), at midcycle surge (MC), and after menopause (PM). Number of women in each group is indicated within parentheses.

**Figure 2.**
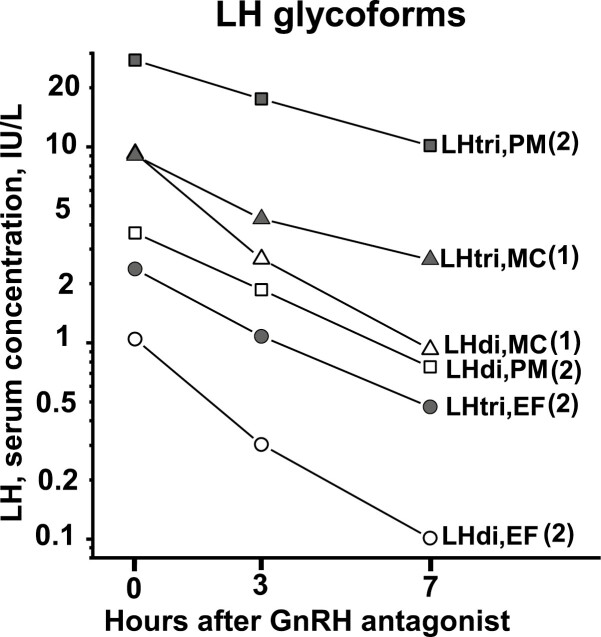
Mean changes of serum concentration of LH glycoforms after GnRH-receptor antagonist, expressed in IU/L, for women in early follicular phase (EF), at midcycle surge (MC), and after menopause (PM). Number of women in each group is indicated within parentheses.

The mean serum concentrations of the glycoforms, before and during the GnRH blockade, for the 3 groups of women are shown in Fig. 1 for FSHtri and FSHtera and in Fig. 2 for LHdi and LHtri. The individual basal serum concentrations of FSHtri and FSHtetra for the 8 women are presented in Table 1 and of those of LHdi and LHtri for the 5 women in Table 2.

**Table 1.** Half-life of circulating FSHtri and FSHtetra glycoforms during blockade with GnRH-receptor antagonist in 8 women

Participants	FSH glycoforms	Serum concentration	FSHtri half-life	FSHtetra half-life
		Basal level IU/L	Minutes	Minutes
Early follicular phase (EF)				
EF1	FSHtri	1.85	317	
EF1	FSHtetra	3.48		914
EF2	FSHtri	1.98	252	
EF2	FSHtetra	3.57		677
EF3	FSHtri	2.64	396	
EF3	FSHtetra	2.36		802
Mean half-life for EF women			322	798
Midcycle phase (MC)				
MC1	FSHtri	3.21	113	
MC1	FSHtetra	5.04		436
MC2	FSHtri	3.22	119	
MC2	FSHtetra	4.38		580
Mean half-life for MC women			116	508
Postmenopausal (PM)				
PM1	FSHtri	15.3	686	
PM1	FSHtetra	68.7		1038
PM2	FSHtri	10.1	274	
PM2	FSHtetra	55.9		939
PM3	FSHtri	12.9	589	
PM3	FSH tetra	64.1		670
Mean half-life for PM women			516	882
Mean ± SEM, n = 8			343 ± 73^*a*^	757 ± 72^*a*^

^*a*^Statistical comparisons of half-lives of FSHtri and FSHtetra, using paired *t* tests: *P *= 0.0003.

**Table 2.** Half-life of circulating serum LHdi and LHtri glycoforms during blockade with GnRH-receptor antagonist in 5 women

Participants	LH glycoforms	Serum concentration	LHdi Half-life	LHtri Half-life
		Basal level IU/L	Minutes	Minutes
Early follicular phase (EF)				
EF1	LHdi	0.71	106	
EF1	LHtri	2.71		133
EF3	LHdi	1.37	84.1	
EF3	LHtri	2.05		107
Mean half-life for EF women			95.0	120
Midcycle phase (MC)				
MC2	LHdi	9.25	90.3	
MC2	LHtri	9.05		139
Postmenopausal (PM)				
PM1	LHdi	3.73	147	
PM1	LHtri	23.5		205
PM2	LHdi	3.51	198	
PM2	LHtri	31.7		235
Mean half-life for PM women			172	220
Mean ± SEM, n = 5			125 ± 21^*a*^	164 ± 24^*a*^

^*a*^Statistical comparison of half-lives of LHdi and LHtri, using paired *t* test: *P* = 0.004.

### Half-life of Gonadotropin Glycoforms Estimated During GnRH Receptor Blockade

The estimated half-life of FSHtri and FSHtetra during GnRH receptor blockade for the 8 women are given in Table 1 and those of LHdi and LHtri for the 5 women in Table 2. The mean values for women: EF, MC, and PM, are also shown in Tables 1 and 2. In each individual, the low-N-glycosylated glycoforms of both FSH and LH had a shorter half-life in the circulation vs the fully N-glycosylated glycoforms. The mean half-life values were 343 and 757 minutes for FSHtri vs FSHtetra (*P* = 0.0003). Two of the 3 of postmenopausal women (PM1 and PM3) had the longest half-life of both FSH glycoforms. The 2 women at midcycle phase (MC1 and MC2) had the shortest half-life of both FSH glycoforms. The mean half-life values were 125 and 164 minutes for LHdi vs LHtri (*P* = 0.004). The 2 postmenopausal women (PM1 and PM2) had the longest half-life of both LH glycoforms. One of the 2 women at early follicular phase (EF3) had the shortest half-life of both LH glycoforms.

In the 5 women for whom results are available for the glycoforms of LH and FSH, the half-lives of the both low and fully N-glycosylated forms of FSH were longer than those of LH (*P* = 0.0008).

## Discussion

Within the gonadotropes FSH and LH beta-subunit polypeptides and the common alpha-subunit polypeptide are first N-glycosylated cotranslationally in the rough endoplasmic reticulum (10). The results of the current study show, that for both FSH and LH, the low-N-glycosylated glycoforms disappeared faster from the human circulation than the fully N-glycosylated forms.

The biological properties of the low- and fully N-glycosylated FSH and LH molecules have been extensively studied (11-18). The same electrophoretic method, as used in the present study for separation of gonadotropin glycoforms in circulation, was previously used for separation of FSH molecules in different pituitary extracts (11). The pituitary FSH molecules with different electrophoretic mobility were then analyzed using in vivo bioassay, in vitro bioassay, and by estimation of disappearance rate in mice. The pituitary FSH molecules with an electrophoretic mobility corresponding to FSHtetra are designated here as “pFSHtetra,” whereas those in which the electrophoretic mobility corresponded to FSHtri are designated “pFSHtri.” Results of these previous studies indicated that pFSHtri had a high activity in the in vitro bioassay that measured estradiol production in cultured Sertoli cells, but a very low or undetectable activity in the in vivo bioassay, which measured ovarian weight in immature mice. In comparison to pFSHtetra, pFSHtri disappeared much faster from the circulation. Similar results for receptor binding and in vitro bioassays have been found for low and fully N-glycosylated forms of highly purified human pituitary LH (14). Taken together, these results suggest that for both gonadotropins, disappearance from the circulation plays a highly significant role in determining in vivo bioactivity. In the current study, we have shown that the degree of glycosylation has effects on the half-lives of circulating FSH and LH in women similar to those observed in purified human pituitary extracts of FSH and LH. The biological effects of circulating FSH and LH in women will thus depend, at least in part, on the relative and dynamic concentrations of the low and fully glycosylated form of each hormone (10).

The fully N-glycosylated form of FSH, FSHtetra, is particularly prominent in the early to mid-follicular phase (19). The in vivo bioactivity of pFSHtetra is greater than that of pFSH in an in vivo bioassay that measured growth of ovarian tissue, and we have now shown that the half-life of FSHtetra is prolonged in women compared with that of FSHtri, as was the case for pFSHtetra vs pFSHtri in a rodent bioassay (19). Taken together, these findings suggest that the glycoform composition of FSH may augment its effect on the early follicular development in the normal menstrual cycle in women. It is of interest that the low-N-glycosylated forms of FSH and LH, FSHtri and LHdi, which thus exhibit the combination of higher in vitro bioactivity with shorter half-lives in the circulation are prominent at the time of the mid-cycle surge in women (19). The preovulatory LH surge is critical for both final oocyte maturation and ovulation; a short half-life may limit the effect of elevated LH on androgen production from nonluteinized theca cells. The increase of FSHtri compared with FSHtetra at the midcycle (19) may reduce the possible effect of FSH on inappropriate recruitment of additional follicles at this time of the cycle.

Both low and fully N-glycosylated forms of FSH and LH, reported in this and other studies, vary in the number of AMS residues, added to the glycans in the Golgi (10). AMS residues include either sialic acid or sulfonated N-acetylgalactosamine (SU), both of which influence the metabolic clearance of the gonadotropins. An increased number of terminal sialic acid residues on the glycans prolongs the survival of the molecules in the circulation (5, 20). In addition, a mannose/sulfonated N-acetylgalactosamine-specific receptor (SU receptor) in the liver removes LH molecules with 2 or more terminal SU residues from the circulation (21, 22), resulting in a shorter LH half-life in women compared with LH isoforms with fewer than 2 SU residues (5). Different degrees of sialylation FSH and sialylation and sulfonation of LH glycoforms will therefore be key factors explaining the differences in half-lives of the low and the fully glycosylated forms of FSH and LH observed in the current study. The dynamic changes of the gonadotropin N-glycosylation, sialylation, and sulfonation observed during the ovarian cycle are essential for achieving successful natural ovulatory cycles (19, 23).

A higher plasma disappearance rate in the mouse for exogenous pituitary FSH from young women compared with that of men and elderly women was reported in 1984 (24). The estimated half-live of human FSH of a 17-year-old woman was 15.8 minutes and that of a 74-year-old woman was 30.8 minutes. These values, measured in the mouse, were about 20 times shorter, when compared with the results of the present study, directly measuring the half-life of the endogenous FSH in the human circulation. This emphasizes the effect of species differences by measuring the metabolic clearance rates of the human FSH in the human compared with that measured in the circulation after treating mice with human FSH.

In conclusion, the half-lives of low and fully N-glycosylated forms of LH were shorter than the corresponding half-lives of FSH when measured in the human circulation. Importantly, the low-N-glycosylated glycoforms of both FSH and LH disappeared from the human circulation faster than the fully N-glycosylated forms. The results emphasize that estimated values for half-life in the circulation of total FSH and total LH will depend on the relative amounts of the 2 glycoforms of each hormone and their individual disappearance rates in circulation, which in turn determine the relative in vivo bioactivities of FSH and LH.

## Data Availability

Some or all data generated or analyzed during this study are included in this published article or in the data repositories listed in References.

## Abbreviations

AMS: anionic monosaccharide
EF: early follicular
MC: midcycle surge
PM: postmenopausal
SU: sulfonated N-acetylgalactosamine
